# 92-year meteorological datasets from 50 meteorological stations in Sinaloa, Mexico with daily, monthly, and yearly resolutions

**DOI:** 10.1016/j.dib.2025.112041

**Published:** 2025-09-09

**Authors:** Zuriel Dathan Mora-Félix, Sergio Alberto Monjardin-Armenta, Jesús Gabriel Rangel-Peraza, Sergio Arturo Rentería-Guevara, Antonio Jesús Sanhouse-García, Yaneth Bustos-Terrones

**Affiliations:** a Universidad Autónoma de Sinaloa, Facultad de Ciencias de la Tierra y el Espacio. Circuito Interior Oriente SN, Cd Universitaria 80040 Culiacán, Sinaloa, Mexico; b Tecnológico Nacional de México/Instituto Tecnológico de Culiacán, División de Estudios de Posgrado e Investigación, Juan de Dios Bátiz 310. Col. Guadalupe 80220 Culiacán, Sinaloa, Mexico; c Laboratorio Nacional CONAHCYT de Tecnologías de la Información Geoespacial para los Sistemas Socioecológicos Resilientes (LaNCTIGeSSR), clave 89. Cerro de Coatepec, Ciudad Universitaria 50110 Toluca de Lerdo, Mexico; d Universidad Autónoma de Sinaloa, Facultad de Ingeniería. Circuito Interior Oriente SN, Cd Universitaria, 80040, Culiacán, Sinaloa, Mexico; e Secretaría de Ciencia, Tecnología, Humanidades, Tecnología e Innovación, TecNM/Instituto Tecnológico de Culiacán, Juan de Dios Bátiz 310. Col. Guadalupe 80220, Culiacán, Sinaloa, Mexico

**Keywords:** Precipitation time series, Machine learning, Forecasting, ETL, Sinaloa

## Abstract

This paper presents a high-resolution long-term meteorological dataset processed from 50 meteorological stations located in Sinaloa, Mexico. These stations are operated by the Comisión Nacional del Agua (CONAGUA), Servicio Meteorológico Nacional (SMN), and Universidad Autónoma de Sinaloa (UAS). A mean of 21,200 records per meteorological station were processed and prepared over the 1933 – 2025 period. The data is available with daily, monthly, and yearly resolutions. These long-term meteorological datasets can be used for water balances, flood and drought simulations, and identifying climate changes and meteorological anthropogenic influences in the study.

Specifications Table

**Table:** 

Subject	Earth & Environmental Sciences
Specific subject area	High temporal resolution measurements of meteorological variables
Type of data	Table
Data collection	The data files were extracted from https://smn.conagua.gob.mx/es/ in txt format. A scanning script is used to extract the data, then the data is pre-processed and transformed into CSV format to identify and remove duplicate records. Subsequently, an empty data filling process is carried out to standardize data formats and ensure data accuracy and consistency. Finally, daily, monthly, and annual resolutions are generated. A repository of data processing algorithms is provided at https://github.com/ZurielMF/86-Y-Metereological_data
Data source location	Data was collected from meteorological stations operated by government Institutions: Comisión Nacional del Agua (CONAGUA), Servicio Meteorológico Nacional (SMN), and Universidad Autónoma de Sinaloa (UAS); located in: Country: México. State: Sinaloa Latitude north, to north 27°02′32″ and south 22°28′02″ Longitude west; to east 105°23′32″ and west 109°26′52
Data accessibility	Repository name: 92-Y-Metereological_data Data identification number: 10.17632/gb8jp62vm5.4 Direct URL to data: https://data.mendeley.com/datasets/gb8jp62vm5/4 The data is released under a CC-BY 4.0 license for research purposes
Related research article	N/A

## 1 Value of the Data

• High-resolution measurements of precipitation, evaporation, maximum and minimum temperatures from 1933 to 2019.
• This dataset provides useful information for understanding the hydro-meteorological events.
• Provides useful information to understand the dynamics of rivers and streams in urban areas and to be able to create protection strategies in the headwaters of river basins.
• It is useful to build machine learning models to forecast high-intensity rainfall and droughts.
• Since data is georeferenced is possible to build geospatial variable analysis.
• Datasets represent diverse geographic zones, along the variations in environmental conditions, and provide data that can be replicated in research conducted in any geographic location.
• The dataset is well-suited for use by the scientific community, including data scientists, artificial intelligence engineers, and specialists in climate behavior analysis, as well as government institutions that require tools for the prevention of events such as droughts and floods.
• The data is published under a CC-BY 4.0 license, enabling the resource to be used for research and technological development.

## 2 Background

There is a growing need for high-resolution long-term climatological and meteorological datasets. These datasets are essential for understanding climate change and its potential impacts on anthropogenic activities, vegetation dynamics, soil water content, and scientific fields [1,2]. For instance, precipitation datasets are extremely useful for evaluating simulations of high-intensity weather events with high-risk level flood [3], developing operational flood, drought monitoring systems [4], and evaluating aquifer outflows and characteristics [5]. High-resolution datasets are also valuable for validating rain gauge observations [6], and precipitation data is essential for analysing the spatial distribution of rainfall in hydrological modelling [7].

Although there is increasing demand for high-resolution precipitation datasets, obtaining accurate and quantitative data remains one of the most significant challenges for scientists developing techniques and constructing datasets. Furthermore, the acquisition and analysis of experimental data are fundamental to understanding hydrological processes and their spatiotemporal variability [8]. Data quality is a critical factor, often compromised by the lack of automated control processes to ensure consistency and reliability. In many cases, datasets contain incomplete records, large gaps, routine record-keeping, or significant alterations, which require transformation and correction procedures to eliminate corrupted data [9].

Additional uncontrollable factors, such as the geographic distribution of meteorological stations, also affect data quality [10]. In México, most of these stations are in remote or difficult access areas. Furthermore, limited infrastructure often prevents real-time transmission of data from the meteorological station to a central server. As a result, data is frequently delayed, unprocessed, or incomplete, leading to fragmented time series. These limitations reduce the availability of long-term, high-quality information.

To address these challenges, this study presents a high-resolution meteorological dataset for the state of Sinaloa. This region represents an agricultural region with diverse climate zones, including coastal, mountain, and desert environments, but lacks a unified, validated, and accessible dataset. The present work fills this gap by providing a comprehensive dataset consisting of over 335,000 daily records from 50 meteorological stations. The primary contribution of this work lies in the meticulous data compilation, rigorous quality control, and homogenization processes, which provide a reliable resource for this specific region. The raw data were transformed into clean, time-series CSV files containing four variables: precipitation, evaporation, and maximum and minimum temperature. Each time series underwent quality control procedures, including decomposition analysis, to ensure data integrity. The resulting dataset is fully compatible with standard data analysis software (e.g., Python, R, MATLAB), enabling detailed hydrological, agricultural, and regional climate modelling.

## 3 Data Description

The repository is organized into five main folders: Raw, Daily, Monthly, Annual, and QC & Metadata. The first four folders (Raw, Daily, Monthly, and Annual) contain time-series data and follow a consistent structure: each includes 50 individual CSV files (one per station) along with a single consolidated file that aggregates data from all stations. Each station's CSV file provides records for precipitation (mm), evaporation (mm), and maximum and minimum temperatures ( °C). The fifth folder (Metadata) contains supporting documentation, including the station metadata table and quality control (QC) summary files.

**Fig. 1:**
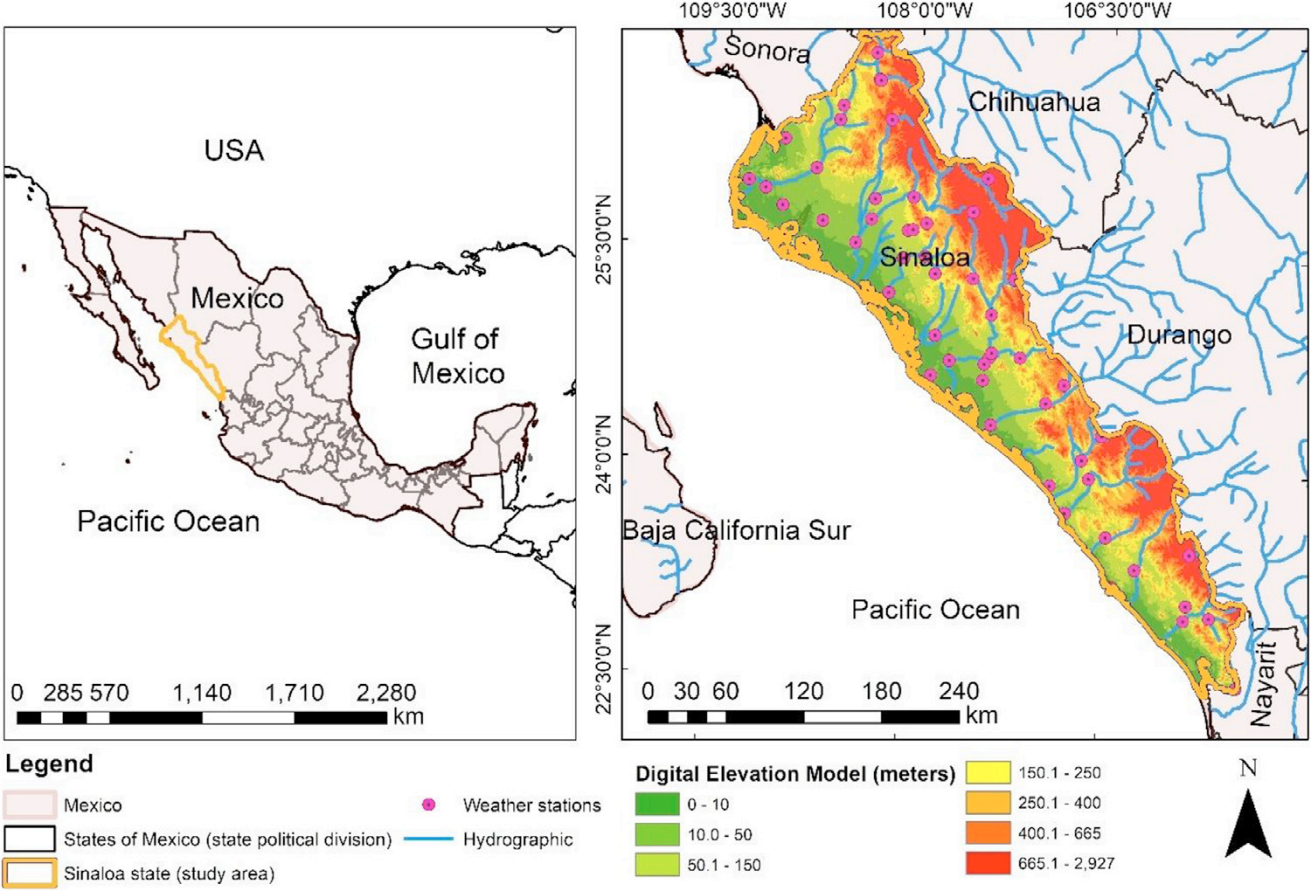
Spatial distribution of the 50 hydrometeorological stations used in this study throughout the state of Sinaloa, Mexico. Each station is represented by its unique code (ID), illustrating the topographic and hydrological context of the measurement network.

The metadata file includes detailed information for each of the 50 meteorological stations, such as official names, station codes, geographic coordinates (latitude and longitude), elevation, instrumentation, and the exact period of record. This information allows users to filter and select stations based on specific criteria. The QC summary file outlines the data quality for each station and variable. It presents the percentage of missing data before the cleaning process and the percentage of data that was subsequently infilled. A detailed description of the dataset is provided in Table 1, Table 2. The geographic distribution of the stations ensures full coverage of the state, as illustrated in Fig. 1.

**Table 1:** Description and measurement equipment for the meteorological variables.

Variable Name	Description	Equipment Used
Precipitation	The precipitation data consists of the raw daily total rainfall published by CONAGUA, measured in mm/day. These measurements are taken once daily at 07:00 local time. The values represent the uncorrected rainfall depth recorded at the station for the preceding 24-hour period.	Manual Hellmann-type precipitation gauge
Evaporation	The evaporation data represent the raw daily values (pan evaporation) published by CONAGUA, measured in millimeters per day (mm/day). These measurements are taken once daily at 07:00 local. The values reflect the direct measurements from the instrument (no pan coefficients have been applied to this data).	Class A evaporation pans with micrometer hook gauge (ETR series)
Temperature max Temperature min	Temperature max and Temperature min represent the raw daily maximum and minimum air temperatures published by CONAGUA, measured in degrees Celsius ( °C). The measurement is taken once daily at 07:00 local time. The values reflect the highest and lowest temperatures recorded over the preceding 24-hour period and are reported without correction.	U-shaped mercury-in-glass Six's thermometer housed within a standardized instrument shelter.

**Table 2:** Description of the station metadata fields included in the dataset files.

Data Name	Description
Station id	The unique identifier for the station.
State	The state where the station is located.
Municipality	The municipality where the station is located.
Status	The current operational status of the station (active or inactive).
Latitude	The geographic latitude of the station.
Longitude	The geographic longitude of the station.

## 4 Experimental Design, Materials and Methods

### 4.1 Data acquisition

The study area is in the state of Sinaloa in northwest Mexico. Sinaloa covers approximately 57,365 km^2^ (Fig. 1). The mean annual precipitation is 790 mm, evaporation is 402.7 mm, and the mean maximum and minimum temperatures are 10.5 °C, 25 °C respectively.

To delineate the area where stations are located, the vector layers of the National Institute of Statistics and Geography (INEGI) were used. The delineation corresponds to stations situated near river boundaries and major dams in the state of Sinaloa; the selected stations represent different climatic zones. The files were extracted from https://smn.conagua.gob.mx/es/ and pre-processed to transform them into CSV format.

The station network covers the diverse geography and climatic zones of Sinaloa (Fig 1). The topography ranges from coastal plains and agricultural valleys to high mountain reliefs. For example, stations 25,022 and 25,032 are located in coastal zones at elevations near 10 m.a.s.l., where high humidity and temperatures are common. In contrast, mountain stations such as 25,074 and 25,093 are situated in the Sierra Madre Occidental at elevations exceeding 1600 m.a.s.l., where cooler and more temperate conditions prevail. This significant topographic gradient influences local climate patterns, and the proximity of some stations to rivers and dams may also affect temperature and humidity readings compared to those in more urbanized areas.

### 4.2 Data processing

**Fig. 2:**
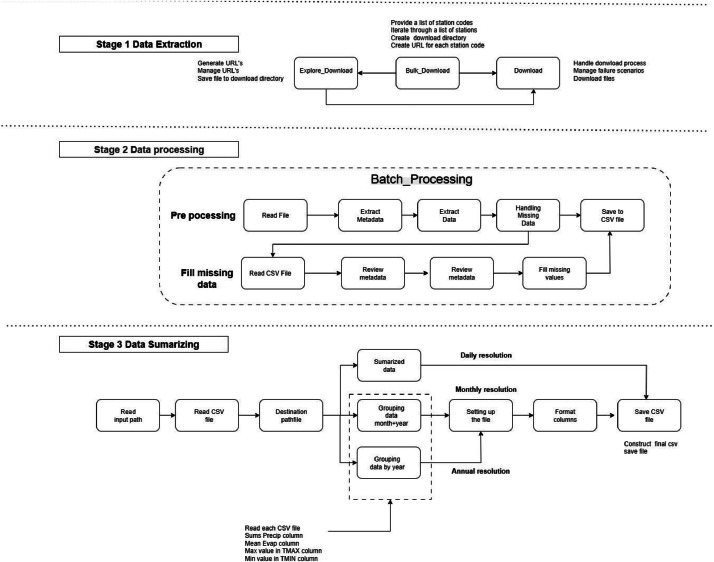
ETL Workflow for obtaining meteorological datasets, including cleaning and transformation Python functions.

The raw data were processed using a custom ETL (Extraction, Transformation, and Loading) pipeline developed in Python. This pipeline standardizes, cleans, and aggregates the data into an analysis-ready format. The complete workflow is summarized in Fig 2.

The process begins by generating download URLs based on a master list of 50 station codes and a defined historical date range. A Python script iterates through these URLs to download the raw data files from the source, storing them in a local directory for subsequent processing stages.

**Fig. 3:**
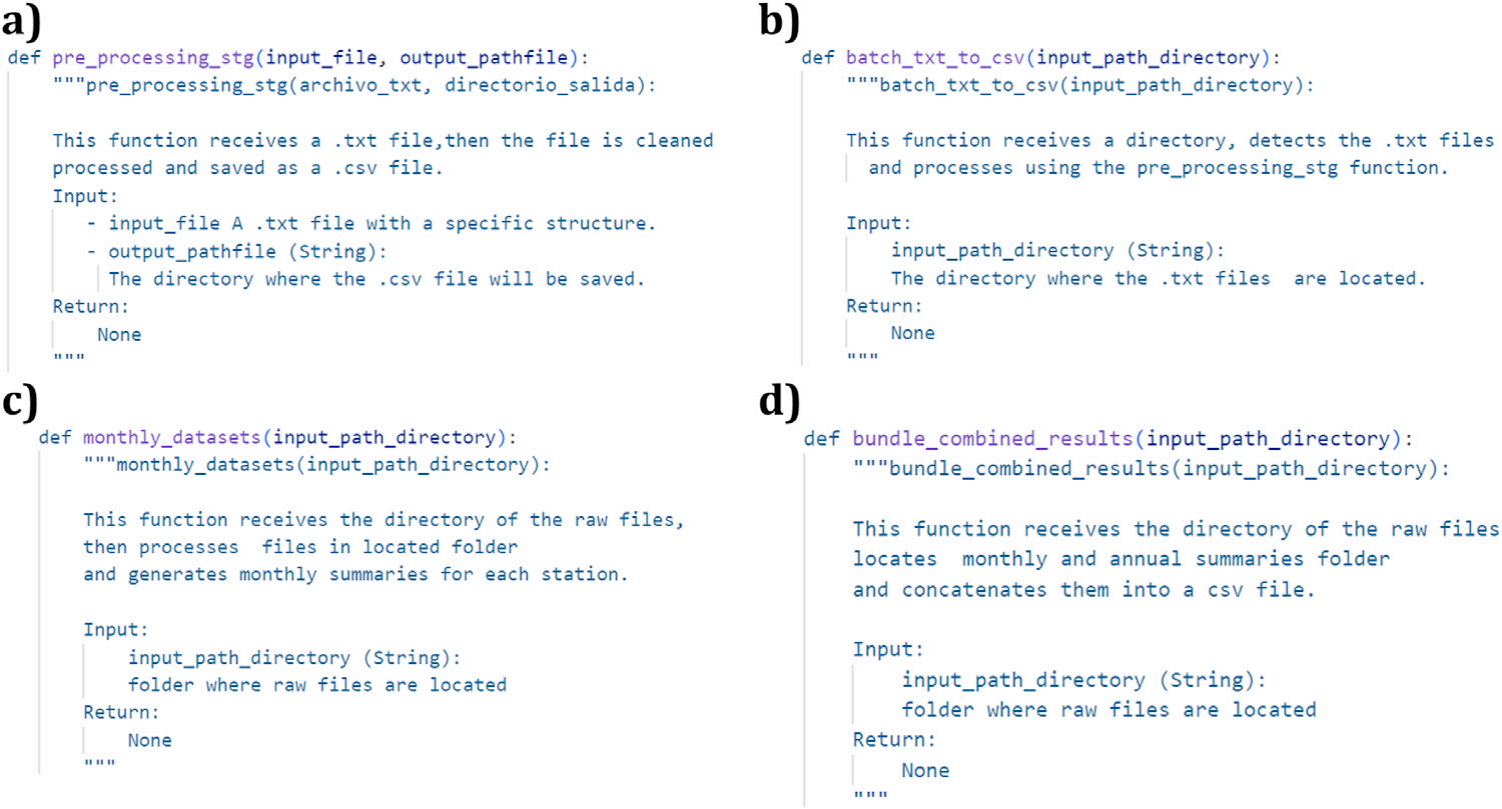
Python code implementing the stages of the data processing pipeline. The stages implemented include: (a) the data cleaning process, (b) batch processing of station data files, (c) generation of monthly and annual resolution data through aggregation, and (d) summarizing the data into final output files.

Then, the raw text files are analysed to remove headers, footers, page numbers, and other metadata labels. The script reformats the data from a fixed-width layout to a delimited structure, replacing blank spaces with commas, and saves the output as a standardized CSV file (Figs. 3a and b). Each row in the cleaned files corresponds to a single day and includes the following columns: date, year, month, precipitation (mm), evaporation (mm), tmax ( °C), tmin ( °C), station_id, latitude, longitude, and elevation (m).

After cleaning, gaps in the daily time series are addressed. The date column is set as the primary index for each file. Missing values in the numeric columns (precipitation, evaporation, tmax, tmin) are infilled using monthly means. For any missing value, this method calculates the long-term historical average for that specific month at that corresponding station and uses this mean as the infilled value. The result is a complete set of daily CSV files.

The cleaned daily files serve as the basis for generating datasets with monthly and annual resolutions. To create the monthly records, the daily data are grouped by year and month, and a specific aggregation function is applied to each variable, as illustrated in Fig 3c. Total monthly precipitation is calculated as the sum of all daily values within that month. Monthly maximum and minimum temperatures are determined by selecting the respective maximum and minimum daily values. The monthly evaporation value is calculated as the mean of the daily records. A similar procedure is used to generate annual records from the monthly data.

**Fig. 4:**
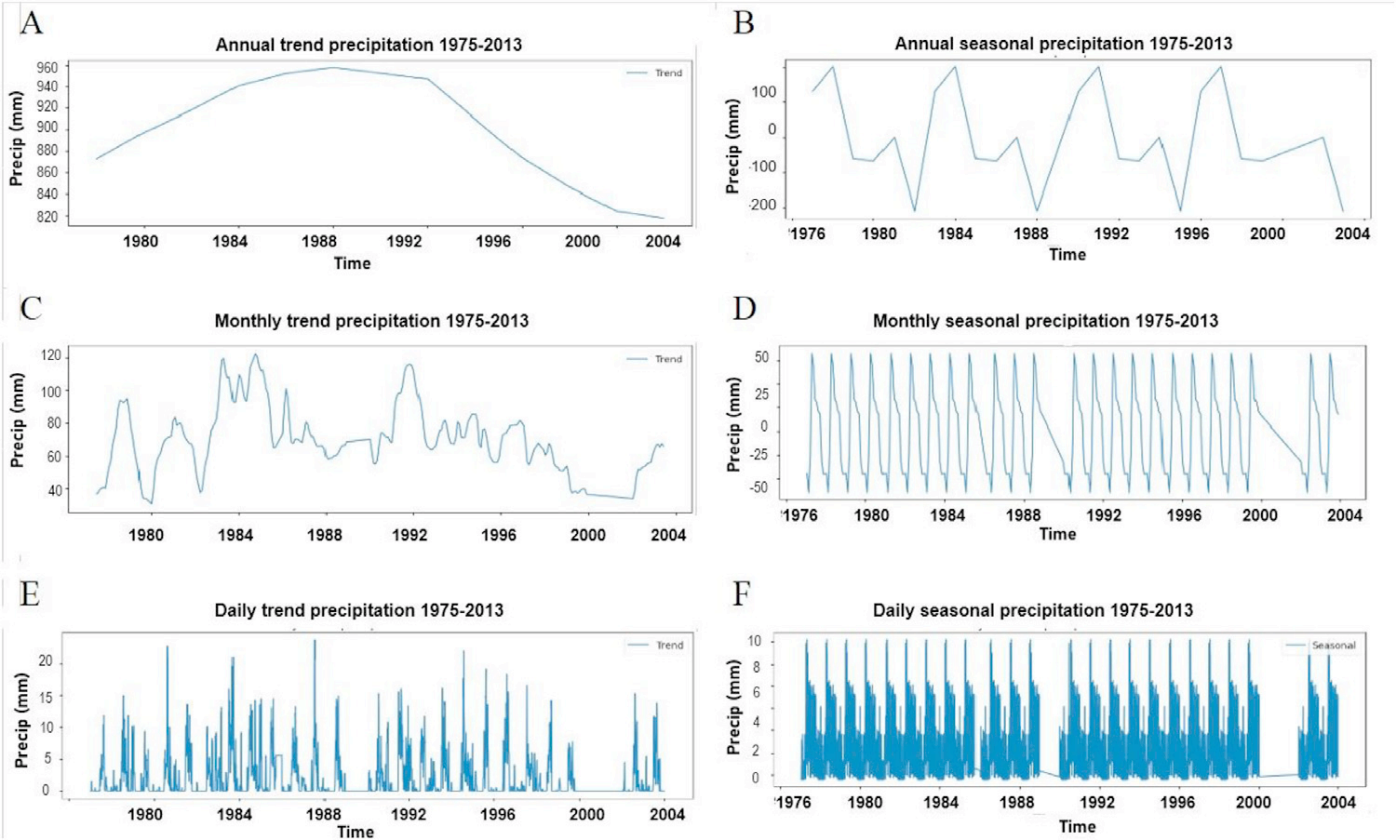
Seasonal-Trend decomposition of the time series for station 25,044. The analysis is presented at daily, monthly, and annual resolutions to illustrate how the time series components (trend, seasonal, and remainder components) are present at different time scales.

The ETL pipeline is executed for all 50 stations, resulting in a set of output files organized by temporal resolution. For each resolution (Daily, Monthly, and Annual), the repository contains 50 individual CSV files, one per station and a single consolidated CSV file, that aggregates data from all 50 stations into a unified table for ease of use in network-wide analyses (Fig. 4d). All output files include columns for station ID, latitude, longitude, and elevation to facilitate geographical analysis. The detailed Python code used to perform this ETL process is available for replication at https://github.com/ZurielMF/86-Y-Metereological_data.

### 4.3 Quality control

A threshold was established for handling missing data to ensure the reliability of aggregated values and long-term statistics. Any given month for a station was considered incomplete and excluded from monthly or annual summaries if it contained more than seven missing daily records for any single variable. This threshold (representing <25 % of a month) prevents the calculation of unrepresentative monthly averages while preserving as much valid daily data as possible.

In addition, a screening process was conducted to identify and flag physically impossible values, such as daily minimum temperatures recorded as higher than maximum temperatures on the same day. These flagged entries were treated as errors and subsequently handled as missing data during processing. However, a different approach was applied to statistically atypical values (outliers). Before removing any extreme data points, particularly for precipitation, values were cross-referenced between neighboring stations and verified against regional meteorological reports. This analysis revealed that most high-precipitation events in the dataset were spatially consistent and corresponded to documented phenomena, such as tropical storms. As a result, these valid atypical values were retained to preserve the dataset's utility for studying climate extremes and to provide an accurate representation of the region’s climatic variability.

### 4.4 Data homogeneity analysis

A homogeneity analysis was conducted to identify any statistically significant break points. For this purpose, the Buishand U test was used, a widely used method for detecting inhomogeneities in climatological data [11]. The Buishand U test is a non-parametric test that evaluates whether the cumulative deviations from the mean of a time series have occurred by chance. The U test statistic is particularly sensitive to a single shift or step-change within the record. First, a subset of 10 stations (20 % of the total dataset) was selected. These stations were chosen to be representative of the different climatic zones (coastal plains, agricultural valleys, and high mountain reliefs) and the wide range of elevations present in the dataset. Then, the Buishand U test was applied individually to the annual time series of precipitation, mean maximum temperature, and mean minimum temperature for each station with a period of record exceeding 30 years. The null hypothesis (H0) for the test is that the time series is homogeneous. A break point was considered statistically significant if the calculated U statistic exceeded the critical value at a significance level of α=0.05.

### 4.5 Gap-Filling method performance

A cross-validation experiment was conducted to assess the performance of the gap-filling algorithm. The procedure was designed to quantify the method's accuracy in predicting missing values across the diverse environmental conditions of the study region. Validation was performed on each selected station for homogeneity analysis. Using segments of the time series with continuous observations, a validation set was created by holding out 10 % of the known data points. These points were treated as artificial gaps, while the remaining 90 % of the observed data in the segment were used as input for the algorithm.

Finally, the gap-filling algorithm was applied to estimate the values of these artificial gaps. Prediction accuracy was evaluated by comparing the algorithm's infilled values against the true observations. The Root Mean Square Error (RMSE) was calculated to assess the performance of the gap-filling method. This procedure was repeated for each key meteorological variable at the selected stations.

### 4.6 Time series validation for stationarity and trends

A validation process was implemented to ensure the suitability of the time-series data for trend analysis and to characterize its structural properties. This process involved an initial time-series decomposition for visual inspection, followed by a suite of formal statistical tests to assess stationarity and detect trend.

The first stage employed the Seasonal-Trend decomposition procedure based on Loess (STL) [12]. The STL method separates a time series into three components: a long-term trend, a periodic seasonal cycle, and a remainder (or residual) component. This decomposition provides a visual assessment of the data's structure and helps identify patterns for subsequent statistical analysis.

Following the decomposition, a series of statistical tests was applied to quantitatively evaluate the properties of the data. Two complementary tests were used to evaluate the stationarity of the time series: the Augmented Dickey-Fuller (ADF) test [13] and the Kwiatkowski-Phillips-Schmidt-Shin (KPSS) test [14]. The ADF test assumes non-stationarity under the null hypothesis, while the KPSS test's null hypothesis assumes the series is stationary. Using both tests provides a more robust assessment than relying on a single method.

The non-parametric Theil-Sen estimator [15] was used to calculate the magnitude (slope) of the trend [16]. The statistical significance of the trend was then evaluated using the Mann-Kendall test [17]. A trend was considered statistically significant if the p-value was <0.05. This combination of the Theil-Sen slope and Mann-Kendall test is widely recommended for determining trends in climatological and hydrological data [18].

## 5 Data validation

### 5.1 Data quality

The application of quality control and gap-filling methods resulted in a complete daily meteorological dataset. The integrity of each data point is documented through a flagging system. A comprehensive summary of these quality control metrics is available in the QC_file.csv file within the data repository.

Analysis of the dataset reveals a high level of overall data quality. On average, over 92 % of daily temperature records (Tmax and Tmin) across all 50 stations are original values. Similarly, 85 % of precipitation records and 81 % of evaporation records are original. This indicates that most of the dataset did not require gap-filling.

The Quality Control (QC) file details the data integrity for each station, showing the percentage of original versus synthetic (infilled) records. For example, Station 25,001 (Ahome) shows a very high percentage of original data, with over 97 % for both Tmax and Tmin. In contrast, station 25,010 (Bamoa) required more substantial gap-filling for its precipitation record, with 68.75 % of the data being original. This level of detail is available for all stations in the QC file, allowing researchers to make informed decisions and select specific station records that best suit the sensitivity of their analysis.

### 5.2 Data homogeneity and gap-filling performance

The data homogeneity analysis using the Buishand U test indicates that most of the long-term station records are free from significant non-climatic breakpoints (Table 3). Over 85 % of the tested annual time series for temperature and precipitation were found to be homogeneous at the 95 % confidence level. A small number of stations were flagged for potential inhomogeneities, which could be related to historical changes in station location or instrumentation.

**Table 3:** Homogeneity analysis and cross-validation results for the gap-filling method.

Station_ID	Climate Zone	Elevation (m)	Variable	RMSE	U test (p-value)
25,173	Coastal	4	Precipitation	2.8073	0.3552
			Evaporation	0.5093	0.0550
			Temp_max	0.6220	0.5785
			Temp_min	0.8414	0.4123
25,093	High mountain	1572	Precipitation	2.7748	0.000*
			Evaporation	0.5799	0.005*
			Temp_max	0.4903	0.7091
			Temp_min	0.6483	0.3441
25,022	Coastal	8	Precipitation	1.5634	0.038*
			Evaporation	0.3757	0.0947
			Temp_max	0.9920	0.019*
			Temp_min	0.7487	0.6738
25,046	Mountain	130	Precipitation	0.0889	0.5906
			Evaporation	0.3774	0.000*
			Temp_max	0.8992	0.000*
			Temp_min	0.7296	0.000*
25,050	Coastal-Valley	9	Precipitation	0.1167	0.1289
			Evaporation	0.3518	0.000*
			Temp_max	0.5972	0.000*
			Temp_min	0.6933	0.0771
25,102	Valley	69	Precipitation	0.2023	0.8179
			Evaporation	0.3591	0.1577
			Temp_max	0.7948	0.4927
			Temp_min	0.8249	0.4284
25,062	Valley	4	Precipitation	0.2023	0.000*
			Evaporation	0.3591	0.0144
			Temp_max	0.7948	0.000*
			Temp_min	0.8249	0.0919
25,074	High mountain	1572	Precipitation	0.2023	0.2357
			Evaporation	0.3550	0.1105
			Temp_max	0.7542	0.0562
			Temp_min	0.8354	0.1236
25,115	Valley	44	Precipitation	1.8675	0.2831
			Evaporation	0.3920	0.014*
			Temp_max	0.7910	0.1841
			Temp_min	0.8681	0.000*
25,183	Mountain	310	Precipitation	0.2022	0.4280
			Evaporation	0.3796	0.2606
			Temp_max	0.7342	0.3765
			Temp_min	0.7994	0.3891

⁎ Data is not homogeneous.

The cross-validation analysis confirms that the selected gap-filling methodology performs accurately under the diverse environmental conditions of the study area. Performance metrics, calculated by comparing the algorithm's predictions against known data, showed low error values for both maximum and minimum temperature variables. The Root Mean Square Error (RMSE) between the observed and the algorithm's infilled values was consistently low, particularly for temperature variables. As summarized in Table 3, the average RMSE for maximum and minimum temperature across the ten validation sites was approximately 1.35 °C and 1.51 °C, respectively. The RMSE for precipitation was higher, reflecting its greater natural variability. Nevertheless, the results confirm that the gap-filling method performs reliably across all variables.

### 5.3 Time series decomposition, trends and stationarity

Fig 4 presents the Seasonal-Trend decomposition (STL) of the time series for station 25,044 (Huites Dam), using data from 1975 to 2013. The decomposition separates the original data into three primary components: the long-term trend, the periodic seasonal pattern, and the irregular remainder. The results reveal a distinct trend, confirming that the original time series is non-stationary. To satisfy the stationarity requirement for further analysis, the trend was removed by applying first-order differencing.

The decomposition provides a detailed characterization of the time series structure. The trend component (Fig. 4A) reveals a non-linear pattern, with an increase from approximately 1980 to 1988, followed by a general decreasing trend. Furthermore, the seasonal component (Fig. 4D) exhibits a clear and repeating annual cycle, consistent with the region's climate, where intense rainfall fluctuations are concentrated in the summer months. Finally, the remainder component (Fig. 4F) represents the data after the trend and seasonal components have been removed and shows no obvious patterns and appears random, confirming that the decomposition successfully captured the primary structures within the time series.

Results of the Mann-Kendall trend test indicate statistically significant trends in temperature and evaporation (Table 4). At both the monthly and annual resolutions, maximum and minimum temperatures exhibit a significant positive (increasing) trend (*p* < 0.05). Conversely, evaporation shows a significant negative (decreasing) trend over the same periods. For precipitation, no statistically significant monotonic trend was detected at either resolution despite the decadal variability observed visually in Fig. 4A (an increase from 1980–1988 followed by a decrease). At the daily resolution, similar results were observed: an increasing trend for temperature and a decreasing trend for evaporation.

**Table 4:** Mann-kendall trend assessment.

Monthly									
var	trend	h	p	z	tau	s	var_s	slope	intercept
PREC	No-trend	False	0.114	0.776	0.164	15	333.666	22.15	777.574
TMIN	increasing	True	0.002	1.458	0.296	27	73.582	0.1666	3.416
TMAX	increasing	True	0.106	1.613	0.329	30	32.405	0.1666	42.916
EVAP	decreasing	True	0.000	−3.381	−0.362	−33	89.593	−0.0778	7.101
**Daily**									

**var**	**trend**	**h**	**p**	**z**	**tau**	**s**	**var_s**	**slope**	**intercept**
PREC	increasing	True	0.026	2.217	0.017	225,752	1036,434	0	0
TMIN	increasing	True	0.006	2.728	0.036	27,752,146	2775,214	0.0001	16.125
TMAX	increasing	True	0.026	2.214	0.020	262,457	1405,131	0	34
EVAP	decreasing	True	1.332	−7.983	−0.075	−947,188	1407,535	−0.0002	6.671
**Annual**									

**var**	**trend**	**h**	**p**	**z**	**tau**	**s**	**var_s**	**slope**	**intercept**
PREC	No-trend	False	0.443	0.766	0.164	15.9	333.666	22.15	777.57
TMIN	increasing	True	0.102	2.567	0.551	43	267.666	0.166666	3.41666
TMAX	increasing	True	3.499	5.094	0.329	30	32.4053	0.16666	42.91666
EVAP	decreasing	True	0.000	−3.381	−0.362	−33	89.593	−0.07786	7.1017

Stationarity of the time series was assessed using the ADF and KPSS tests, with results presented in Table 5. The analysis confirms that the time series at the daily and monthly resolutions are seasonal but stationary after differencing. For the original data, the ADF test failed to reject the null hypothesis of non-stationarity. However, after applying first-order differencing, the p-value became statistically significant (*p* < 0.05), confirming stationarity.

**Table 5:** KPSS and ADF stationarity assessment.

Serie	Daily analysis				Monthly analysis				annual analysis			
	ADF	p-value	KPSS	p-value	ADF	p-value	KPSS	p-value	ADF	p-value	KPSS	p-value
Original(evap)	−2.194	0.208	0.113	0.1	−2.719	0.070	0.224	0.1	−0.759	0.830	0.493	0.043
diff=1	−9.952	0.000	0.064	0.1	−4.095	0.000	0.047	0.1	0.342	0.979	0.403	0.075
Original(precip)	−10.03	0.000	0.232	0.1	−3.564	0.006	0.159	0.1	−0.543	0.883	0.232	0.100
diff=1	−8.314	0.000	0.500	0.04	−9.798	0.000	0.075	0.1	1.235	0.996	0.305	0.100
Original(tmax)	−3.402	0.010	0.033	0.1	−3.247	0.017	0.029	0.1	0.939	0.993	0.463	0.049
diff=1	−10.87	0.000	0.019	0.1	−9.472	0.000	0.018	0.1	−76.99	0.000	0.357	0.095
Original(tmin)	−1.367	0.597	0.048	0.1	−2.513	0.112	0.027	0.1	0.939	0.993	0.463	0.049
diff=1	−8.176	0.000	0.010	0.1	−9.926	0.000	0.011	0.1	−76.99	0.000	0.357	0.095

In contrast, the annual resolution data were found to be non-stationary. The p-value for the ADF test remained above the 0.05 significance level even after differencing. This result was corroborated by the KPSS test, where the null hypothesis of stationarity was rejected for the annual time series. This lack of stationarity is consistent with the long-term trend visually identified in the decomposition plot (Fig. 4A).

## Limitations

The period of record varies considerably across the 50 stations. The dataset comprises data from 1993 to 2025; however, not all stations were active throughout the entire study period. Researchers conducting long-term trend analysis should carefully consider the number of active stations available at any given time, as detailed in the station_metadata.csv file.

The weather station network provides broad coverage across the Sinaloa state, but the spatial distribution of the stations is not uniform. Station density is lower in higher elevation areas, which may limit the accuracy of spatial interpolations in these specific regions.

Another potential limitation of this dataset concerns the long-term homogeneity of some station records. A homogeneity test revealed that while more station records are consistent, a few exhibit potential anomalies likely caused by undocumented station relocations or equipment changes. Since the exact cause could not be confirmed, the data were not adjusted. Therefore, researchers should be cautious when using these flagged stations for long-term trend studies.

Finally, the evaporation data included in this dataset were obtained from pan evaporimeters. Users should be aware that these measurements are subject to known uncertainties and may not directly represent landscape-scale evapotranspiration. These data are best suited for analysing relative trends rather than serving as an absolute measure of evaporative water loss.

## Ethics Statement

The work described in this article meets all the ethical requirements for publication in Data in Brief. This research did not involve any human participants or animal subjects. The data presented were sourced from institutional meteorological records and do not contain any personally identifiable information.

## CRediT Author Statement

**Zuriel Mora-Felix:** Data processing, Script writing, Original draft, Data processing, writing document; **Jesus Gabriel Rangel-Peraza:** Data processing, Data cleaning, Conceptualization, Methodology, Supervision, Writing-review & editing; **Sergio Monjardin-Armenta**: Formal anaylsis, Methodology, Supervision, Validation; **Sergio Renteria-Guevara:** Investigation, Supervision; **Jesus Antonio Sanhouse-Garcia:** Data processing; **Yaneth Bustos-Terrones:** Project administration, writing document

## Data Availability

Mendeley Data86-Year Meteorological Datasets from 50 Meteorological Stations in Sinaloa, Mexico with Daily, Monthly, and Yearly Resolutions (Original data).
